# The complete plastome sequence of *Clematis guniuensis* (Ranunculaceae), a new plant species endemic to China

**DOI:** 10.1080/23802359.2019.1704662

**Published:** 2020-01-07

**Authors:** Ming Jiang, Junfeng Wang, Huijuan Zhang

**Affiliations:** a Zhejiang Provincial Key Laboratory of Plant Evolutionary and Conservation, College of Life Science, Taizhou University, Jiaojiang, China;; b Lishui Institute of Forestry, Lishui, China

**Keywords:** *Clematis guniuensis*, plastome, phylogenetic analysis

## Abstract

*Clematis guniuensis* is a new plant species in China, which distributes in both provinces of Zhejiang and Anhui with extremely small population. The whole chloroplast (CP) genome was assembled by NovoPlasty using high-throughput sequencing data generated by an Illumina Hiseq X Ten platform. The circular chloroplast genome is 159,682 bp in length, and comprises a large single copy of 79,461 bp, a small single copy of 49,156 bp, and two inverted repeats of 31,064 bp each. The CP genome contains 137 genes, including 89 protein-coding genes, eight rRNA genes, 36 tRNA genes, and four pseudogenes. All the pseudogenes were *infA*, *rpl32*, *ycf1*, and *ndhK*. Phylogenetic analysis results indicated that *C. guniuensis* was sister to *C. trichotoma*, with a support rate of 100%.

The genus *Clematis* belongs to the family Ranunculaceae which has a number of ornamental plant species in the world. The genus is comprised of about 300 species, and 147 of them distribute in China with more than 90 endemic species (Wu et al. 2011). *C. guniuensis* is a perennial herb growing up to 3–5 m, with woody vines, 3-lobed leaflets, and light green petals. It is a new wild plant species found recently in both provinces of Anhui and Zhejiang, and its population size and distribution range are extremely small (Wang et al. 2019). In this study, we *de novo* assembled the complete chloroplast genome of *C. guniuensis*, and a phylogenetic tree was generated to reveal its relationship with other *Clematis* species.

Fresh leaves were collected at an altitude of 933 m in Daruokeng (28°09′08″N, 119°51′36″E), Lishui, Zhejiang Province, China. The voucher specimen (CHS2018032) is deposited at the Molecular Biology Laboratory in Taizhou University. Total genomic DNA was extracted from leaf tissues following the cetyltrimethylammonium bromide (CTAB) method described by Doyle and Doyle (1987) for high-throughput sequencing library construction. In total, about 5.9 Gb raw reads were obtained on an Illumina Hiseq X Ten platform (Illumina, CA, USA), and after filtering, clean reads were used for CP genome assembly by NOVOPlasty (Dierckxsens et al. 2017). The CP genome was then annotated by applying an online tool namely Dual Organellar GenoMe Annotator (DOGMA) (Wyman et al. 2004). Prediction of tRNAs was carried out by two tRNA detection programs, tRNAscan-SE and ARAGORN (Lowe and Eddy, 1997; Laslett and Canback, 2004), respectively.

Our results showed that the complete chloroplast genome of *C. guniuensis* (GenBank accession: MN527334) was 159,682 bp in size. The plastome contains a large single copy (LSC) and a small single copy (SSC) divided by a pair of inverted repeat (IR) regions. The sizes of LSC, SSC, and IR regions were 79,461 bp, 49,156 bp, and 31,064 bp, respectively. The CP genome is consisted of 137 genes, these include 89 protein-coding genes, 8 rRNA genes, 36 tRNA genes, and four pseudogenes. The four pseudogenes are *infA*, *rpl32*, *ycf1*, and *ndhK*. Ten protein-coding genes (*atpF*, *ndhA*, *ndhB*, *petB*, *petD*, *rpl2*, *rpl16*, *rpoC1*, *rps12*, *rps16*) and six tRNA genes (*trnA-UGC*, *trnG-UCC*, *trnI-GAU*, *trnK-UUU*, *trnL-UAA*, and *trnV-UAC*) contain one intron, while another three protein-coding genes, *clpP*, *rps12*, and *ycf3*, contain two introns.

To understand the phylogenetic relationship of *C. guniuensis* with other *Clematis* species, CP genome sequences of ten *Clematis* plants and *Naravelia pilulifera* (KY213888) were downloaded from NCBI. The ten plants were *C. alternate* (NC_039577), *C. trichotoma* (NC_043828), *C. acerifolia* (NC_039844), *C. macropetala* (NC_041477), *C. repens* (NC_039578), *C. fusca* var. *coreana* (KM652489), *C. loureiroana* (NC_039690), *C. uncinate* (NC_039846), *C. brachyura* (NC_042793), and *C. terniflora* (KJ956785). A maximum likelihood phylogenetic tree was constructed by PhyML 3.1 (Guindon et al. 2010), using *N. pilulifera* as an outgroup. Our results indicated that *C. guniuensis* was sister to *C. trichotoma*, with a support rate of 100% (Figure 1).

**Figure 1.**
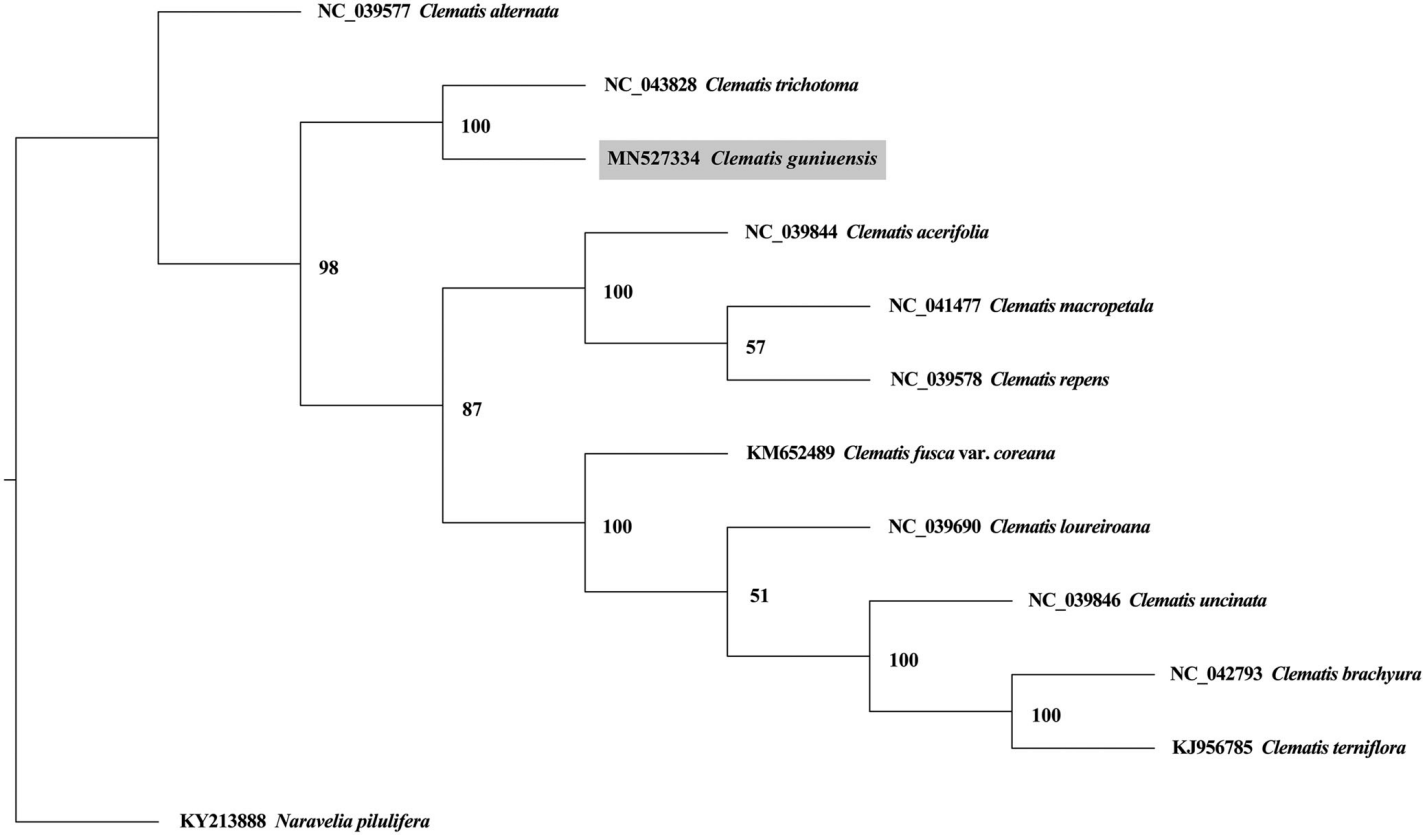
A maximum likelihood tree based on the complete chloroplast genome sequences of *Clematis guniuensis* and other *Clematis* species, with *Naravelia pilulifera* (Ranunculaceae) as an outgroup. The numbers next to nodes indicate bootstrap support values.
